# Anemia in inflammatory bowel disease: prevalence, differential diagnosis and association with clinical and laboratory variables

**DOI:** 10.1590/1516-3180.2014.1323568

**Published:** 2014-04-14

**Authors:** Rodrigo Andrade Alves, Sender Jankiel Miszputen, Maria Stella Figueiredo

**Affiliations:** I Postgraduate Student, Division of Gastroenterology, Department of Medicine, Universidade Federal de São Paulo (Unifesp), São Paulo, Brazil; II PhD. Head of Intestine Sector, Division of Gastroenterology, Department of Medicine, Universidade Federal de São Paulo (Unifesp), São Paulo, Brazil; III PhD. Professor, Discipline of Hematology, Department of Medicine, Universidade Federal de São Paulo (Unifesp), São Paulo, Brazil

**Keywords:** Anemia, Inflammatory bowel diseases, Prevalence, Association, Diagnosis, differential, Anemia, Doenças inflamatórias intestinais, Prevalência, Associação, Diagnóstico diferencial

## Abstract

**CONTEXT AND OBJECTIVES:**

Anemia is the most frequent extraintestinal complication of inflammatory bowel disease. This study aimed to: 1) determine the prevalence of anemia among patients with inflammatory bowel disease; 2) investigate whether routine laboratory markers are useful for diagnosing anemia; and 3) evaluate whether any association exists between anemia and clinical/laboratory variables.

**DESIGN AND SETTING:**

Cross-sectional at a federal university.

**METHODS:**

44 outpatients with Crohn's disease and 55 with ulcerative colitis were evaluated. Clinical variables (disease activity index, location of disease and pharmacological treatment) and laboratory variables (blood count, iron laboratory, vitamin B_12_ and folic acid) were investigated.

**RESULTS:**

Anemia and/or iron laboratory disorders were present in 75% of the patients with Crohn's disease and in 78.2% with ulcerative colitis. Anemia was observed in 20.5% of the patients with Crohn's disease and in 23.6% with ulcerative colitis. Iron-deficiency anemia was highly prevalent in patients with Crohn's disease (69.6%) and ulcerative colitis (76.7%). Anemia of chronic disease in combination with iron deficiency anemia was present in 3% of the patients with Crohn's disease and in 7% of the patients with ulcerative colitis. There was no association between anemia and disease location. In ulcerative colitis, anemia was associated with the disease activity index.

**CONCLUSIONS:**

Most patients present iron laboratory disorders, with or without anemia, mainly due to iron deficiency. The differential diagnosis between the two most prevalent types of anemia was made based on clinical data and routine laboratory tests. In ulcerative colitis, anemia was associated with the disease activity index.

## Abstract (pt)

**CONTEXTO E OBJETIVOS:**

Anemia é a mais frequente complicação extraintestinal na doença inflamatória intestinal. Este estudo objetivou: 1) determinar a prevalência de anemia em portadores de doença inflamatória intestinal; 2) investigar se os marcadores laboratoriais de uso rotineiro são úteis para o diagnóstico da anemia; 3) avaliar se existe associação entre anemia e variáveis clínico-laboratoriais.

**TIPO DE ESTUDO E LOCAL:**

Estudo transversal em uma universidade federal.

**MÉTODOS:**

Foram avaliados 44 pacientes ambulatoriais com doença de Crohn e 55 com retocolite ulcerativa. Foram investigados aspectos clínicos (índice de atividade da doença, localização da doença e tratamento farmacológico) e laboratoriais (hemograma, ferrocinética, vitamina B_12_ e ácido fólico).

**RESULTADOS:**

Anemia e/ou anormalidades na ferrocinética estavam presentes em 75% dos pacientes com doença de Crohn e em 78,2% dos pacientes com retocolite. Anemia foi observada em 20,5% do grupo com doença de Crohn e em 23,6% do grupo com retocolite. Anemia por deficiência de ferro predominou entre os pacientes com doença de Crohn (69,6%) e com retocolite (76,7%). Anemia de doença crônica associada à anemia ferropriva estava presente em 3% dos pacientes com doença de Crohn e em 7% daqueles com retocolite. Na retocolite, a anemia estava associada com o índice de atividade da doença.

**CONCLUSÕES:**

A maioria dos pacientes apresentava alterações na ferrocinética com ou sem anemia, principalmente decorrente da ferropenia. O diagnóstico diferencial entre os dois tipos mais prevalentes de anemia foi baseado nos dados clínicos e nos testes laboratoriais de rotina. Anemia estava associada com o índice de atividade na retocolite.

## INTRODUCTION

Inflammatory bowel diseases (IBD) comprise two main distinct clinical entities: Crohn's disease (CD) and ulcerative colitis (UC). CD is a chronic transmural inflammation that can affect any segment of the digestive tract and is associated with numerous extraintestinal manifestations.^1^ UC is the result of chronic inflammation of the colon, affecting one or more of its segments. Generally, the distal segments are affected and all viscera and the distal ileum may eventually be involved.^2^ ^,^ ^3^

For a long time, anemia has been considered to be an inevitable complication of CD and UC and its treatment has only recently become a specific objective for the management of such individuals.^4^ Gasché et al.^5^ showed that 33% of patients with CD had anemia [defined as hemoglobin (Hb) < 12 g/dl]. Another study reported a prevalence of anemia (Hb < 10 g/dl) of 26% in CD and of 37% in UC.^6^

Iron deficiency is the most common type of anemia and is generally underdiagnosed. Chronic and occult blood loss through the gastrointestinal tract is the most frequent cause of iron deficiency.^7^ Malabsorption of iron is uncommon, unless the proximal digestive tract is extensively affected by CD.^4^ ^,^ ^8^

Anemia of chronic disease (ACD) is the second most frequent type of anemia in IBD.^4^ ^,^ ^9^ This condition is characterized by relative deficiency of erythropoietin which is slightly greater, although still very little in relation to the degree of anemia, when compared with anemia of the same intensity observed in other etiologies.^6^ ^,^ ^9^ Cytokines such as interleukin 1 (IL-1), tumor necrosis factor (TNF) and interferon-γ are produced in larger amounts by monocytes and mononuclear cells of the intestinal lamina propria.^6^ IL-1 and interferon-γ decrease the production of erythropoietin and inhibit erythropoiesis. TNF and IL-1 increase the production of ferritin, thus reducing the amount of iron available for erythropoiesis.^10^ Moreover, some *in vitro* and *in vivo* studies have demonstrated that these cytokines induce resistance to erythropoietin in erythroid precursors.^6^ ^,^ ^11^ ^,^ ^12^ In 2000, a peptide synthesized in the liver, called hepcidin, was isolated from plasma. Expression of this peptide is induced by lipopolysaccharide and IL-6. This peptide increases the capacity of reticuloendothelial cells in the duodenal crypts to take up and retain iron and decreases dietary iron absorption.^13^ ^,^ ^14^ Hepcidin exerts its biological function upon binding to the only known cellular iron exporter, ferroportin, thereby leading to ferroportin internalization and degradation. This event blocks transfer of the absorbed iron from the duodenal enterocyte into the circulation and, in parallel, causes retention of iron inside macrophages and monocytes.^15^

Other causes of anemia, such as vitamin B_12_ deficiency, generally occur when resection of the ileum exceeds 60 cm. Inadequate ingestion, malabsorption and use of sulfasalazine or methotrexate contribute towards emergence of megaloblastic anemia due to folic acid deficiency. In addition, drugs such as immunosuppressants, mesalazine and sulfasalazine can cause bone marrow suppression.^4^ ^,^ ^16^ Some studies have demonstrated that treatment of anemia has a positive impact on the quality of life of patients with IBD.^17^ ^,^ ^18^

The differential diagnosis between iron-deficiency anemia (IDA) and ACD is important since only the former will respond solely to replacement therapy. Moreover, evidence indicates that administration of iron to patients with chronic inflammation may trigger an increase in production of oxygen free radicals, thus propagating tissue injury.^8^ ^,^ ^10^ ^,^ ^19^ ^,^ ^20^

The high prevalence of anemia among patients with IBD observed in the international literature encouraged the present investigation conducted on Brazilian patients with IBD. Although this abnormality is highly common, its diagnosis and treatment have not received the same attention by physicians as have other complications such as arthritis and osteopathy.^20^ ^-^ ^22^ Another objective was to determine whether or not it is possible to establish the etiology of anemia using noninvasive complementary tests that are widely available in clinical practice. In addition, we evaluated whether the presence of these abnormalities is associated with clinical and/or laboratory activity, location of the disease and medications used.

## OBJECTIVES

1. To determine the prevalence of anemia in a group of Brazilian patients with inflammatory bowel disease;
2. To investigate whether routine laboratory markers are useful for the etiological diagnosis of anemia;
3. To evaluate the association between anemia and clinical/laboratory variables.

## METHODS

Forty-four patients with CD and 55 with UC, who were being regularly followed up at the intestinal sector of the gastroenterology outpatient clinic of a federal university hospital in São Paulo, Brazil, were studied. The diagnoses of Crohn's disease and ulcerative colitis were based on the usual clinical criteria.^1^ ^,^ ^2^ Patients were included as they visited the clinic and if they met the inclusion criteria, from March 2001 to May 2002. Patients with known hemoglobinopathies, myelodysplastic syndromes, sideroblastic anemia, kidney failure, liver disease and hypothyroidism, and patients requiring hospital admission were excluded.

The study protocol was approved by the hospital's ethics committee, and all patients or their legal representatives signed an informed consent form to participate in the study.

The Harvey and Bradshaw index^23^ and the Truelove and Witts index^24^ were used to quantify clinical disease activity in patients with CD and UC, respectively. The disease location in CD was defined by the Vienna classification of Crohn's disease.^25^ Blood was collected during regular pharmacological treatment (aminosalicylates, corticosteroids, antibiotics and/or immunomodulators). Iron supplementation was discontinued for at least one week before blood collection in patients taking oral iron and for one month in those receiving parenteral iron.^26^ The following laboratory parameters were evaluated: complete blood count, erythrocyte sedimentation rate, reticulocyte count, serum iron, transferrin concentration, ferritin, bilirubins, lactate dehydrogenase, vitamin B_12_ and folic acid. The transferrin saturation was calculated according to the formula of Turati et al.^27^ Hemoglobin electrophoresis was carried out in the cases of patients with microcytosis and normal serum iron.^28^ ^-^ ^30^

Anemia was defined as Hb levels of less than 13 g/dl in males and less than 12 g/dl in females.^31^ Iron deficiency was defined based on reduced serum ferritin concentration (< 30 ng/ml) and/or low transferrin saturation (< 16%). In the presence of clinical or biochemical evidence of inflammation, the lower limit of serum ferritin consistent with normal iron stores was 100 ng/dl. The diagnostic criteria for ACD were serum ferritin > 100 ng/ml and transferrin saturation < 16%, in the presence of biochemical or clinical evidence of inflammation. The definition of functional iron deficiency (FID) was the same, but with Hb levels within the normal range. The association between IDA and ACD was characterized by transferrin saturation < 16% and serum ferritin between 30 and 100 ng/dl.^32^ Macrocytosis was defined as a mean corpuscular volume > 95.1 fl in males and > 98.3 fl in females.

The term hematological abnormality was used to define alterations in iron laboratory values (relating to iron deficiency or to functional iron deficiency observed in chronic diseases), with or without associated red blood cell abnormalities. This term was also used when referring to macrocytosis. 

By accepting an alpha of 5% with statistical power of 90% and anemia as the main outcome, a sample of 37 cases for each group of diseases was estimated. A virtual calculator was used, which was obtained through the following site: http://www.lee.dante.br/pesquisa/amostragem/amostra.html.

### Statistical analysis 

The statistical analysis was performed using the Statistical Package for the Social Sciences (SPSS) for Windows software, versions 10.0.1 and 13.0 (SPSS, Chicago, IL, USA). Numerical variables were expressed as the mean, median, standard deviation and range. Nominal variables were reported as absolute (n) and relative (%) frequencies. Student's t test was used to compare numerical variables between the two groups of patients. Nominal variables were compared using the χ^2^ test or Fisher's exact test, when necessary. The level for rejection of the null hypothesis was set at 0.05 or 5%.

## RESULTS

**Demographic and clinical characteristics**

Women predominated in both the CD and the UC group, with no significant difference between the groups. No difference in mean age was observed between the two groups. With respect to the location of the disease, the ileal-colonic (L3) (47.7%) and ileal (L1) (40.9%) forms predominated in patients with CD, whereas pancolitis (34.7%) and rectosigmoid involvement (30.9%) were more frequent in patients with UC (P < 0.01). The prevalence of individuals who had previously undergone intestinal and/or colon resection was higher among CD patients (61.4%), whereas only two patients (3.6%) in the UC group had undergone resection (P < 0.01). Active disease was more prevalent among patients of the UC group than in those of the CD group (P < 0.01) (Table 1).

**Table 1.** Demographic and clinical characteristics of the patients with Crohn's disease (CD) and ulcerative colitis (UC)

	CD	UC	P-value
Gender			
Male	15 (34.1%)	22 (40.0%)	
Female	29 (65.9%)	33 (60.0%)	
**Total**	**44 (100%)**	**55 (100%)**	P = 0.67*
Age (years)			
Mean ± SD	41.6 ± 10.7	43.8 ± 14.0	
Median (range)	43 (22-70)	43 (17-81)	
95% confidence interval	38.4 - 44.9	40.0 - 47.2	P = 0.40†
Disease location			
Ileum (LI)‡	18 (40.9%)	-	
Ileum-colon (L3)‡	21. (47.7%)	1 (1.8%)	
Colon (L2)‡	4 (9.1%)	7 (12.7%)	
Pancolitis (L2)‡	1 (2.3%)	19 (34.7%)	
Rectosigmoid	-	17 (30.9%)	
Rectum	-	11 (20%)	
**Total**	**44 (100%)**	**55 (100%)**	**P < 0.01§**
Surgical resection			
None	17 (38.6%)	53 (96.4%)	
Distal ileum	9 (20.5%)	-	
Ileum	2 (4.5%)	-	
Right colon	4 (9.1%)	-	
Ileum and right colon	12 (27.3%)	-	
Total colectomy	-	2 (3.6%)	
**Total**	**44 (100%)**	**55 (100%)**	**P < 0.01§**
Disease activity			
Active	11 (25%)	47 (85.5%)	
Remission	33 (75%)	8 (14.5%)	
**Total**	**44 (100%)**	**55 (100%)**	P < 0.01*

* Fisher's exact test

† Student's t test

‡ Vienna Classification for CD

2 test.

### Etiology of hematological abnormalities and prevalence of anemia

No difference in the etiological factor of hematological abnormalities was observed between patients with CD and those with UC (P = 0.77). In the CD group, 25% of the patients did not present hematological abnormalities. The most frequent types of hematological abnormalities were iron deficiency (ID) (without anemia) in 45.4% and iron deficiency anemia (IDA) in 24.2%. In the UC group, 21.8% of the patients did not present any hematological abnormality. ID was present in 53.5%, IDA in 23.2 % and ID + FID in 9.3% (Table 2). Hemoglobin levels below the lower limit of normal were observed in 22 patients, including 9 with CD and 13 with UC (P = 0.36) (Table 3).

**Table 2.** Etiology of hematological abnormalities in patients with Crohn's disease (CD) and ulcerative colitis (UC) (n = 76)

	CD	UC	Total
ID	15 (45.4%)	23 (53.5%)	38 (50.0%)
ID + FID	3 (9.1%)	4 (9.3%)	7 (9.2%)
FID	3 (9.1%)	2 (4.6%)	5 (6.6%)
IDA	8 (24.2%)	10 (23.2%)	18 (23.7%)
IDA + ACD	1 (3.0%)	3 (7.0%)	4 (5.2%)
Macrocytosis	3 (9.1%)	1 (2.3%)	4 (5.2%)
**Total**	**33 (100%)**	**43 (100%)**	**76 (100%)**

ID = iron deficiency (without anemia)

FID = functional iron deficiency (without anemia)

IDA = iron-deficiency anemia

ACD = anemia of chronic disease

χ2 test. P = 0.77.

**Table 3.** Prevalence of anemia in male and female patients with Crohn's disease (CD) and ulcerative colitis (UC) (n = 22) CD U

	CD	UC	Total
Male (Hb < 13 g/dl)	1 (4.5%)	4 (18.2%)	5 (22.7%)
Female (Hb < 12 g/dl)	8 (36.4%)	9 (40.9%)	17 (77.3%)
**Total**	**9 (40.9%)**	**13 (59.1%)**	**22 (100%)**

Hb = hemoglobin. Fisher's exact test

P = 0.36

### Associations 

Activity versus hematological abnormalities

No association between clinical disease activity and hematological abnormalities was observed in patients with CD (P = 0.47) (Table 4). In the UC group, 93% (40/43) of the patients with hematological abnormalities presented mild or moderate activity. Three of the eight individuals in remission showed hematological abnormality (P = 0.03) (Table 5).

**Table 4.** Association between clinical disease activity and hematological abnormalities in patients with Crohn's disease (n = 44)

Hematological abnormality	Activity	Remission	Total
None	1 (2.3%)	10 (22.7%)	11 (25%)
ID	3 (6.8%)	12 (27.3%)	15 (34.1%)
ID + FID	1 (2.3%)	2 (4.5%)	3 (6.8%)
FID	1 (2.3%)	2 (4.5%)	3 (6.8%)
IDA	3 (6.8%)	5 (11.4%)	8 (18.2%)
IDA + ACD	1 (2.3%)	-	1 (2.3%)
Macrocytosis	1 (2.3%)	2 (4.5%)	3 (6.8%)
**Total**	**11 (25%)**	**33 (75%)**	**44 (100%)**

ID = iron deficiency (without anemia)

FID = functional iron deficiency (without anemia)

IDA = iron-deficiency anemia

ACD = anemia of chronic disease

χ2 test; P = 0.47.

**Table 5.** Association between clinical-laboratory activity and hematological abnormalities in patients with ulcerative colitis (n = 55)

Hematological abnormality	Activity			Total
	Remission	Mild	Moderate	
None	5 (9.1%)	5 (9.1%)	2 (3.6%)	12 (21.8%)
ID	3 (5.5%)	12 (21.8%)	8 (14.5%)	23 (41.8%)
ID + FID	-	3 (5.5%)	1 (1.8%)	4 (7.3%)
FID	-	1 (1.8%)	1 (1.8%)	2 (3.6%)
IDA	-	2 (3.6%)	8 (14.5%)	10 (18.2%)
IDA + ACD	-	-	3 (5.5%)	3 (5.5%)
Macro	-	-	1 (1.8%)	1 (1.8%)
**Total**	**8 (14.5%)**	**23 (41.8%)**	**24 (43.6%)**	**55 (100%)**

ID = iron deficiency (without anemia)

FID = functional iron deficiency (without anemia)

IDA = iron-deficiency anemia

ACD = anemia of chronic disease

**Macro:** = macrocytosis. χ2 test

P = 0.03

*Location of the disease versus hematological abnormalities*

No association between the location of the disease and the presence or absence of hematological abnormalities was observed in the CD group (P = 0.54) or in the UC group (P = 0.41) (Table 6).

**Table 6.** Association between disease location and hematological abnormalities in patients with Crohn's disease (CD) and ulcerative colitis (UC)

Location	CD		UC	
	With hematological abnormalities	Without hematological abnormalities	With hematological abnormalities	Without hematological abnormalities
Ileum	13 (29.5%)	5 (11.4%)	–	–
Ileum-colon	17 (38.6%)	4 (9.1%)	1 (1.8%)	–
Colon	2 (4.5%)	2 (4.5%)	6 (10.9%)	1 (1.8%)
Pancolitis	1 (2.3%)	–	17 (30.9%)	2 (3.6%)
Rectosigmoid	–	–	11 (20.0%)	6 (10.9%)
Rectum	–	–	8 (14.5%)	3 (5.5%)
Total	33 (75%)	11 (25%)	43 (78.2%)	12 (21.8%)

*Pharmacological treatment versus hematological abnormalities*

No association between the type of pharmacological treatment and the presence or absence of hematological abnormalities was observed in either group (P = 0.32 in CD and 0.73 in UC) (data not shown).

## DISCUSSION

Although a high prevalence of anemia among patients with IBD has been recognized in the international literature for decades, many services do not possess a protocol for its investigation. Measurement of hemoglobin levels and serum iron alone is insufficient: hemoglobin measurements only allow diagnosing of the third and last stage of IDA, while iron measurement does not allow differentiation between IDA and ACD. In a study conducted in the 1980s involving hospitalized patients, only 11 of the 29 patients initially diagnosed with IDA had iron deficiency and most of them actually presented ACD.^33^ One important fact is that iron deficiency is 2 to 5 times more frequent than IDA.^31^ Thus, it is possible that the prevalence of iron deficiency is underestimated, especially if measurement of serum ferritin is not part of the routine diagnosis.

The present study demonstrated that most patients with IBD had some type of hematological abnormality, with no difference in the distribution of these abnormalities between CD and UC (Table 2). A cross-sectional study does not provide information about the mean duration of episodes of anemia. However, analysis of these patients provides an overview of the burden of anemia in a population with IBD during the natural history of the disease. Among the 22 patients with hemoglobin levels below the normal limit, 9 had CD and 13 had UC. The prevalence of anemia was similar in the CD and UC groups (Table 3). Two aspects of this result should be highlighted: first, the predominance of female patients (77.3%), which probably reflects their predominance in the present sample as a whole; second, the lack of a difference between CD and UC, which might be due to the relatively small number of anemic subjects. Therefore, the prevalence of anemia observed in this study, i.e. 20.5% among patients with CD and 23.6% among patients with UC (data not shown), was lower than that reported in some other studies.^5^ ^,^ ^6^ However, in a review published by Wilson et al.,^34^ the prevalence of anemia ranged from 10.2% to 72.7% among outpatients with CD and from 8.8% to 66.6% among patients with UC.

In the CD group, 23/33 patients (69.6%) with hematological abnormalities presented some degree of iron deficiency. The same was observed for 33/43 patients (76.7%) of the UC group (Table 2). Coexistence of ID + FID was found in 9.1% of patients with CD and in 9.3% of the UC group. However, this difference was not statistically significant. According to a review by Kulnigg and Gasche,^7^ the prevalence of iron deficiency in patients with CD ranged from 36% to 90% according to the sample studied and to the definition of iron deficiency.

No difference in the prevalence of ACD was observed between patients with CD and those with UC (Table 2). ACD associated with IDA was present in 3.0% of the patients with CD (1/33) and in 7.0% of the patients with UC (3/43). Although ACD is the second leading cause of hematological abnormalities in IBD, studies reporting the true prevalence of this complication are scarce. In the study by Schreiber et al.,^6^ the prevalence of ACD was 15% among patients with CD and 10% among those with UC. Lakatos et al.^35^ reported prevalences of 17.7% and 9.6%. In the present study functional iron deficiency was presented in 9.1% with CD and in 4.6% of the patients with UC.

Coexistence of IDA and ACD has been reported in the literature mainly for patients with rheumatoid arthritis. Ahluwalia et al.^36^ observed this association in 64% of women over the age of 70 years. A recent study among patients with IBD indicated that the combination IDA/ACD was the third most frequent pathological condition underlying anemia.^11^ In the present investigation, this condition was observed in only one patient with CD and in three patients with UC (Table 2). We believe that this low prevalence is due to the difficulty in detecting this association in routine tests. Nevertheless, when both types of hematological abnormality occur together, microcytosis is more frequent and anemia tends to be more intense. The ratio between soluble transferrin receptor concentration and log ferritin level might be a useful parameter. A ratio < 1 suggests ACD, whereas a ratio > 2 suggests iron deficiency coexisting with ACD.^14^

Four patients who were not anemic had macrocytosis, including three women with CD, one of them also presenting iron depletion, and one man with UC (Table 2). The mean corpuscular volume was 111.9 fl for patients with CD and 97.3 fl for the patient with UC (data not shown). Lakatos et al.^35^ found macrocytosis in 4.0% of patients with CD and UC, a prevalence slightly lower than that observed in the present study (5.2%) (Table 2). One CD patient had a normal serum level of vitamin B_12_ and low folic acid level. The second CD patient presented a low level of vitamin B_12_ and normal level of folic acid. The last patient showed normal levels of vitamin B_12_ and folic acid. The UC patient had low levels of vitamin B_12_ and folic acid (data not shown).

One of the objectives of this investigation was to evaluate associations between hematological abnormalities and some clinical characteristics of IBD. No association was observed between hematological abnormalities and the clinical activity index in CD, probably because most patients (75%) were in remission. Interestingly, there was a high prevalence of hematological abnormalities in patients in remission (69.7%) and in those presenting clinical activity (90.9%) (Table 4). In contrast, an association between clinical-laboratory activity and the presence of hematological abnormalities was found in the UC group (Table 5), such that 93% of the patients with hematological abnormalities presented mild or moderate activity, whereas three of the eight patients in remission presented hematological abnormalities (iron deficiency as etiology). The other patients with iron deficiency, as well the patients with ACD, FID and the patient with macrocytosis, presented mild or moderate activity. Since the Truelove-Witts index^24^ is influenced by Hb, this result is not unexpected. Gasché et al.^5^ studied a group of 49 patients with CD and did not observe any difference in the activity index between patients with and without anemia. Similar results were obtained in the present study, although those authors used the CDAI and we chose to use the Harvey-Bradshaw index. However, other authors^6^ ^,^ ^11^ have demonstrated an association between the severity of anemia and disease activity indices in CD and UC patients.

Another intriguing finding was the possibility of an association between the location of IBD and hematological abnormalities. No such association was observed in the CD group or in the UC group (Table 6). In the latter group, 39.5% of the patients with hematological abnormalities had pancolitis, whereas less extensive areas such as the rectosigmoid predominated among patients without hematological abnormalities.

No association was observed between the type of pharmacological treatment and the presence of hematological abnormalities (data not shown). This assessment was carried out because anemia is considered to be an adverse effect of some drugs used for the treatment of IBD, especially sulfasalazine, which inhibits folic acid absorption, and azathioprine, which can cause pancytopenia and macrocytosis.

There may be a tendency to look upon anemia as an unavoidable accompaniment to IBD. Only in recent years has its correction been highlighted as a specific therapeutic aim in these patients. It should not be assumed that some level of anemia is a normal finding in IBD patients. Thus, the World Health Organization definitions of anemia apply to patients with IBD.^21^ For patients in remission or with mild disease, laboratory screening should be performed every 6 or 12 months. The minimum workup should include complete blood count, serum ferritin, serum iron, transferrin concentration, transferrin saturation, lactate dehydrogenase and reticulocyte count. In outpatients with active disease, such measurements should be performed at least every three months. Patients at risk of vitamin B_12_ or folic acid deficiency (e.g. small bowel disease or resection) need proper surveillance. Serum levels of vitamin B_12_ and folic acid should be measured at least annually, or if macrocytosis is present.^32^ However, even the results from recently available tests such as measurement of soluble transferrin receptor indicate that there is a need for other laboratory tests that are able to distinguish between IDA and ACD. In this regard, standardization of the different kits used for measurement of soluble transferrin receptor, or even the soluble transferrin receptor/log ferritin ratio, might be a solution for this problem.

## CONCLUSIONS

Anemia and/or iron disorders were important systemic complications in the group of IBD patients studied here. Anemia was observed in 20.5% of the patients with Crohn's disease and in 23.6% with ulcerative colitis. Anemia of chronic disease in combination with iron deficiency anemia was present in 3% of the patients with Crohn's disease and in 7% of the patients with ulcerative colitis. The differential diagnosis between the two most prevalent types of anemia, IDA and ACD, was made based on clinical data and routine laboratory tests. However, no marker that exclusively helped with or confirmed this diagnosis could be identified. There was no association between anemia and disease location and between anemia and pharmacological treatment. In ulcerative colitis cases, anemia was associated with the disease activity index.
